# Supplementary material for: Inhibition of HIV-1 gene transcription by KAP1 in myeloid lineage
Source: Sci Rep. 2021 Jan 29;11:2692. doi: 10.1038/s41598-021-82164-w (PMC7846785; doi:10.1038/s41598-021-82164-w)

Inhibition of HIV-1 gene transcription by KAP1 in myeloid lineage

Amina AIT AMMAR[1,2,3], Maxime BELLEFROID[3], Fadoua DAOUAD[1], Valérie MARTINELLI[4], Jeanne VAN ASSCHE[1], Clémentine WALLET[1], Anthony RODARI[3], Marco DE ROVERE[1], Birthe FAHRENKROG[4], Christian SCHWARTZ[1], Carine VAN LINT[3*], Virginie GAUTIER[2*] and Olivier ROHR[1*]

1. Université de Strasbourg, UR 7292 DHPI, FMTS, IUT Louis Pasteur, 1 Allée d'Athènes 67300 Schiltigheim, France

2. Center for Research in Infectious Diseases (CRID), School of Medicine and Medical Science (SMMS), University College Dublin (UCD), Dublin, Ireland

3. Service of Molecular Virology, Institute for Molecular Biology and Medicine (IBMM), Université Libre de Bruxelles (ULB), Gosselies, Belgium

4. Laboratory Biology of the Nucleus, Institute for Molecular Biology and Medicine, Université Libre de Bruxelles, Charleroi 6041

Supplementary figure 1

Figure 1

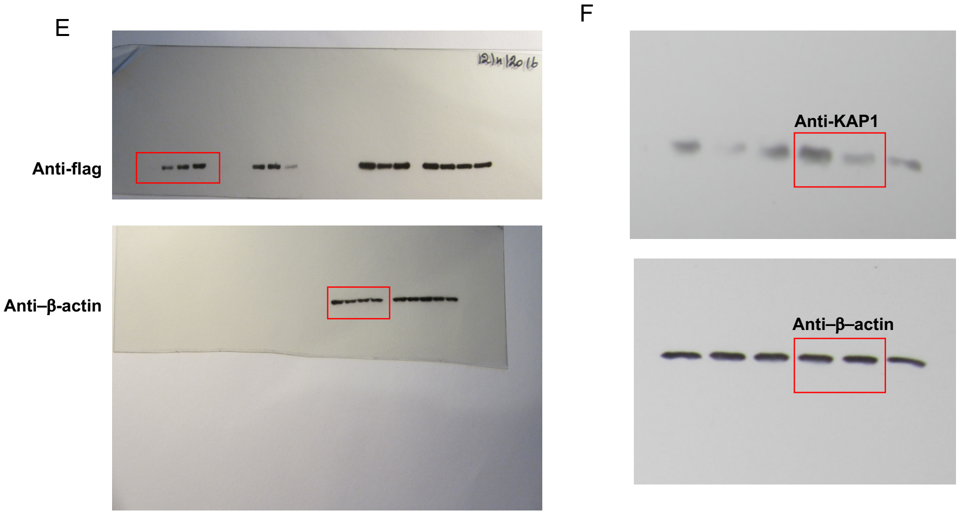

Supplementary figure 2

Figure 3A

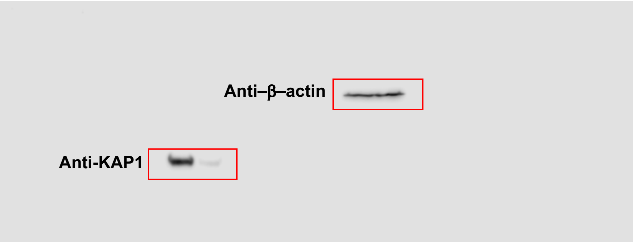

Supplementary figure 3

Figure 4

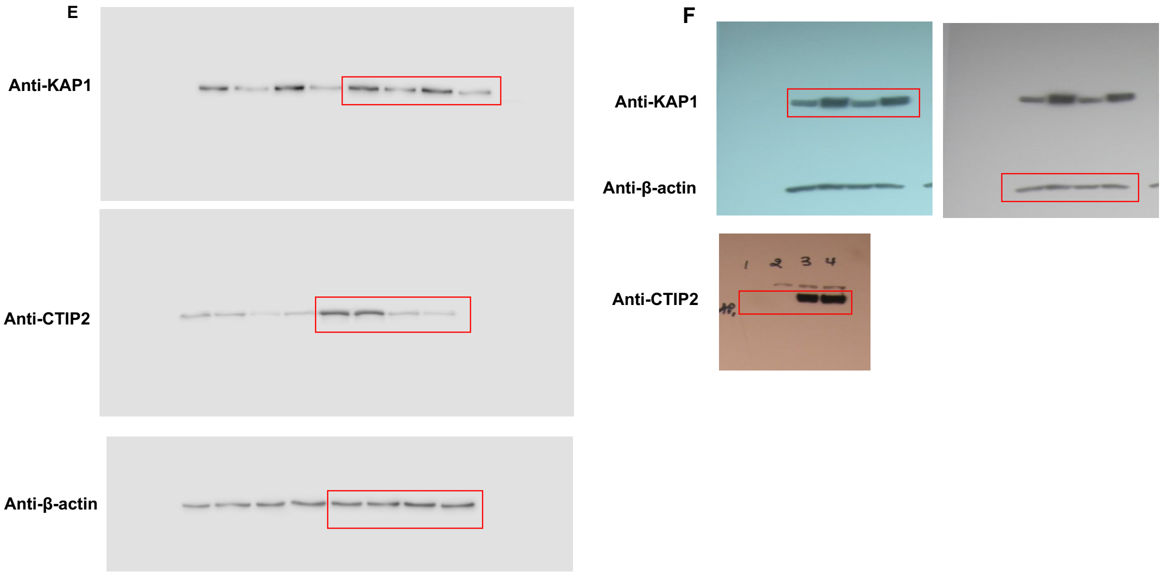

Supplementary figure 4

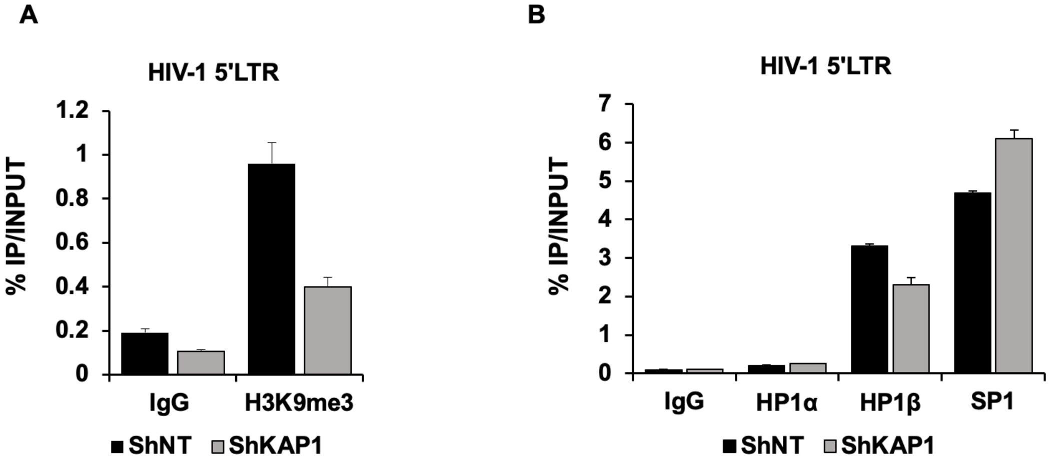

KAP1 depletion leads to reduced heterochromatin marks at the HIV-1 promoter

(A, B) The HIV-1 infected monocytic THP89 cells stably transduced either with non-targeting shRNA (indicated as shNT) or shKAP1 were subjected to ChIP-qPCR experiments using the indicated antibodies and the primers targeting the HIV-1 5' LTR. Values are presented as percentages of immunoprecipitated DNA compared to the input DNA (% IP/INPUT) and are representative of biological duplicates.

Supplementary figure 5

Figure 5B

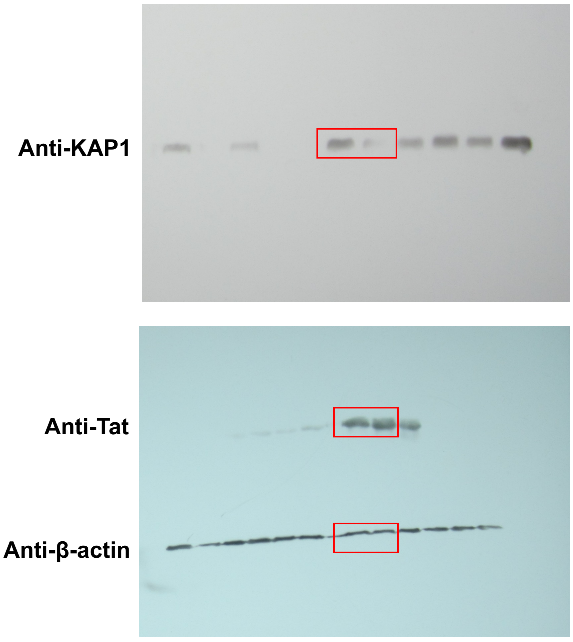

Figure 5C

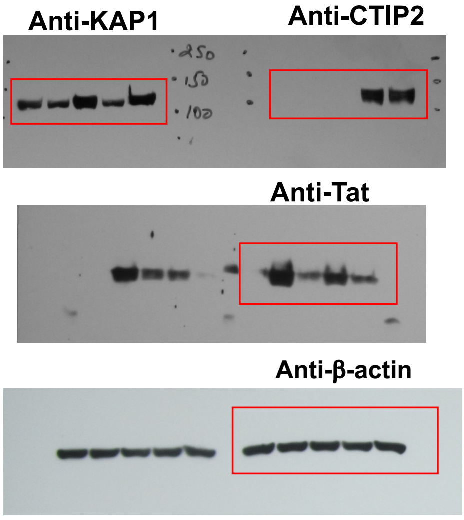

Figure 5D

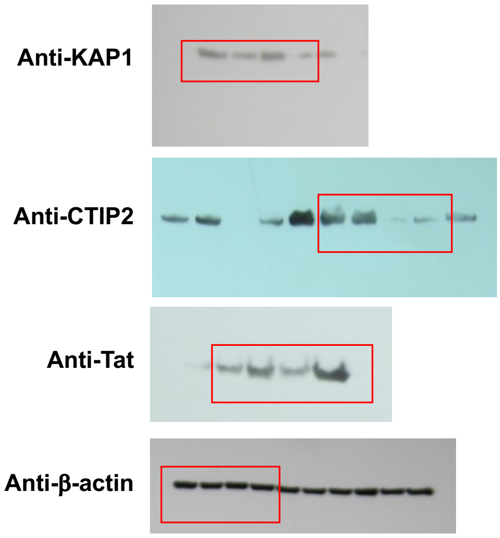

Supplementary figure 6

Figure 6A

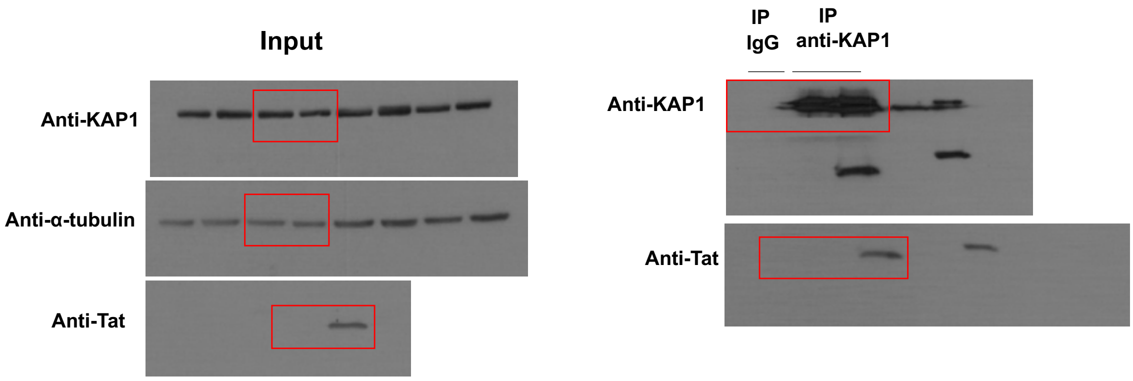

Figure 6B

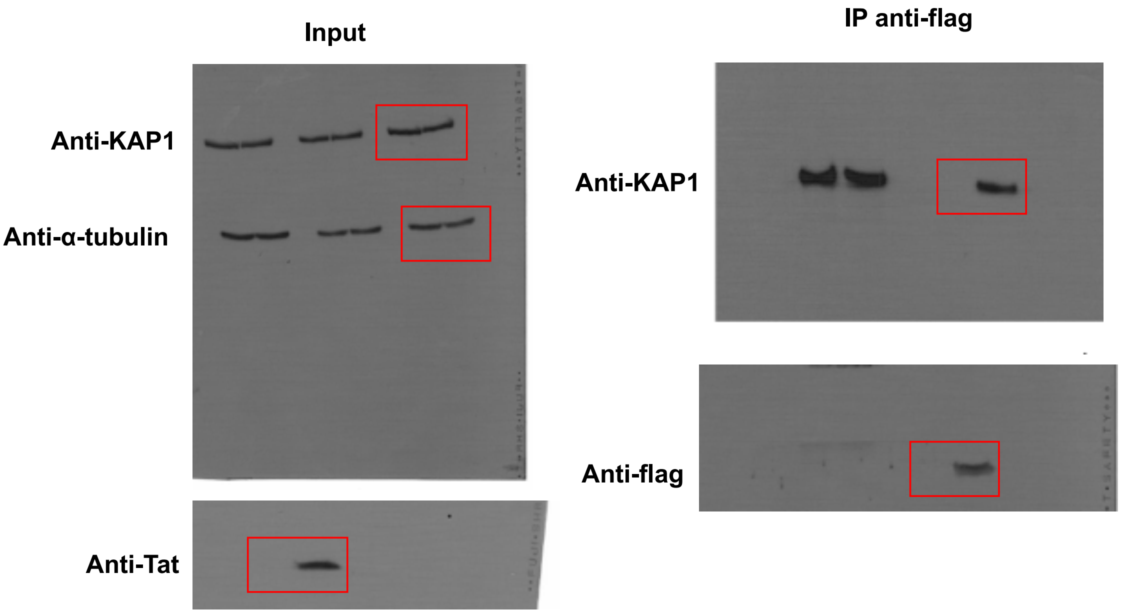

Supplementary figure 7

Figure 7A

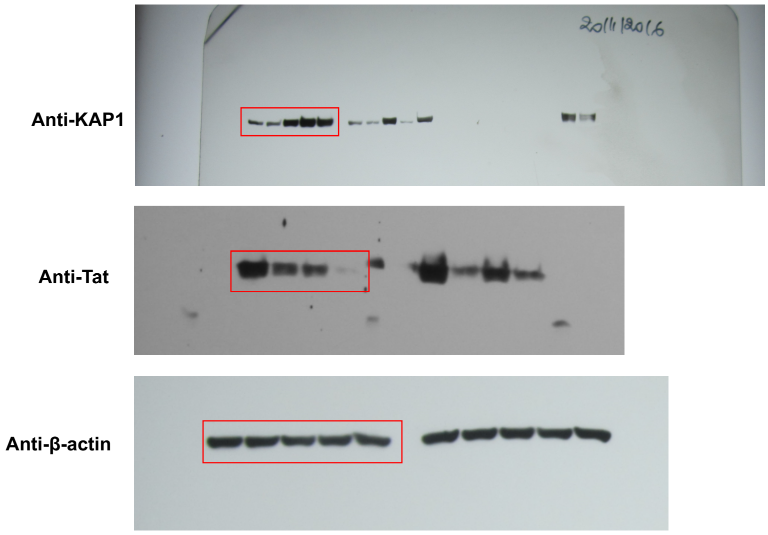

Figure 7B

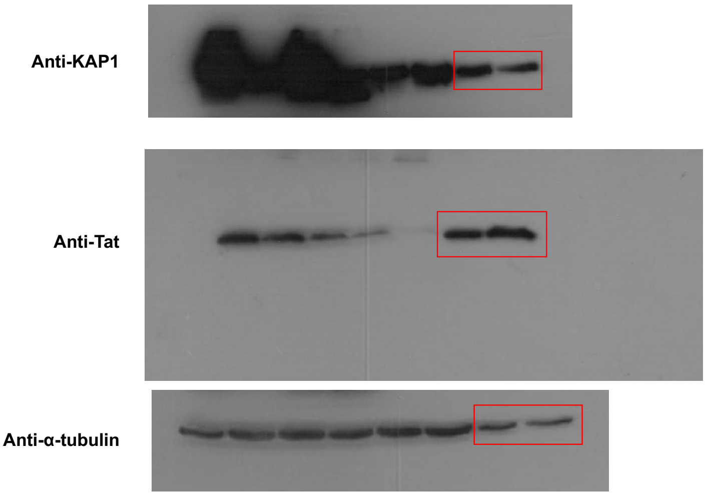

Figure 7D

Anti-flag KAP1

Anti-β-actin

Anti-Tat

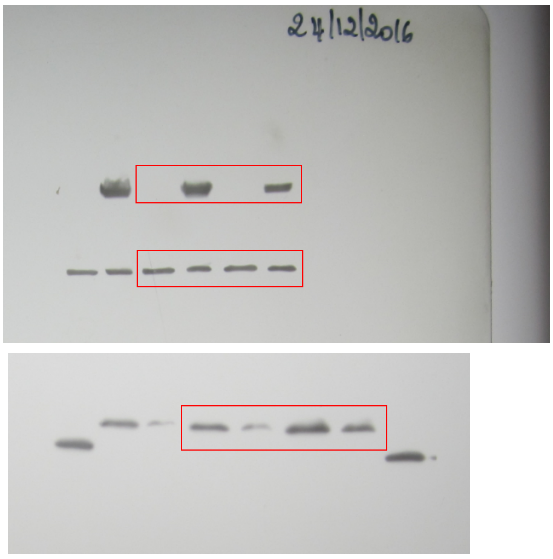

Figure 7F

Anti-KAP1

Anti-Tat

Anti-β-actin

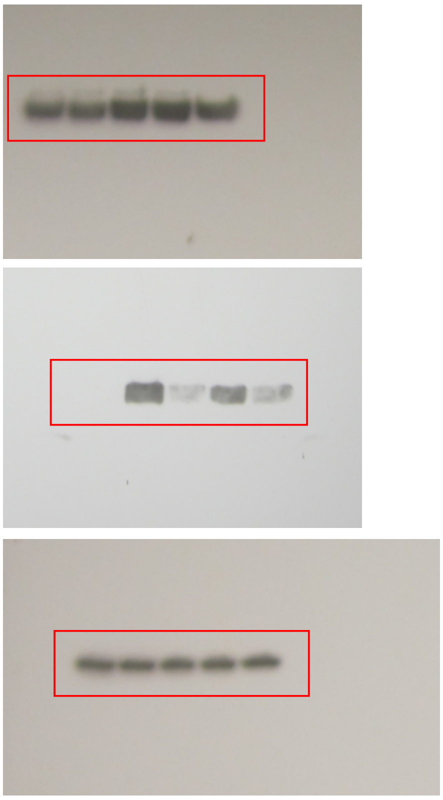

Supplementary figure 8

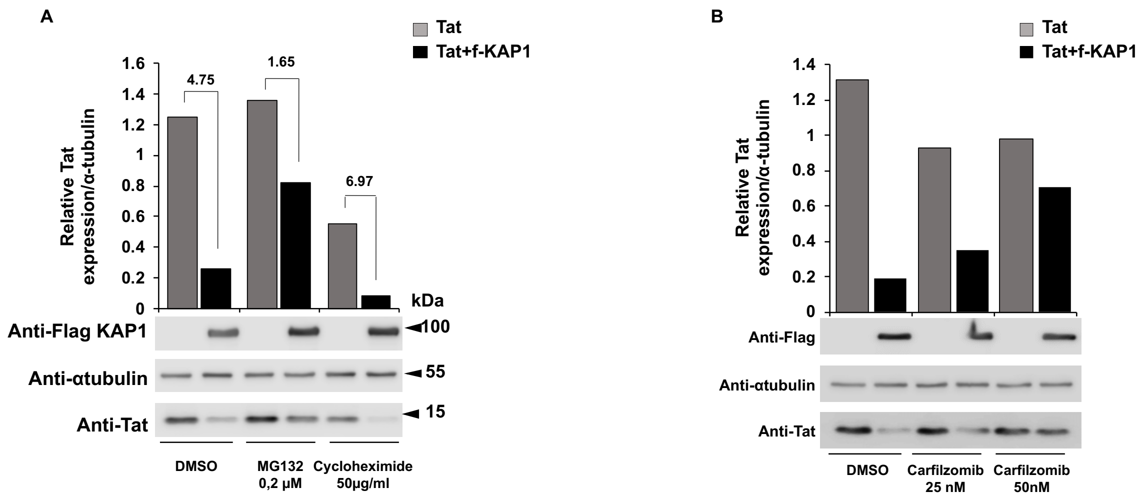

Inhibition of protein synthesis does not impact proteasomal degradation of Tat by KAP1

HEK cells cultured in a 6-well plate were transfected with expression vectors encoding Tat alone or in combination with flag-KAP1 (A, B). 4h post-transfection, the cells were treated with 0.2 μM of MG132 for 20 hours or 50 μg/ml cycloheximide for 4h (A). 4h post-transfection the cells were treated with either 25 nM or 50nM of carfilzomib for 20 hours (B). DMSO treated cells were used as control (A, B). 24h post-transfection cells were lysed, and total protein extracts were analyzed by western blot for the presence of Tat and KAP1. Tat expression levels were normalized to α-tubulin expression, using image J software. The results are representative of two independent experiments.

Supplementary figure 9

Figure 8A

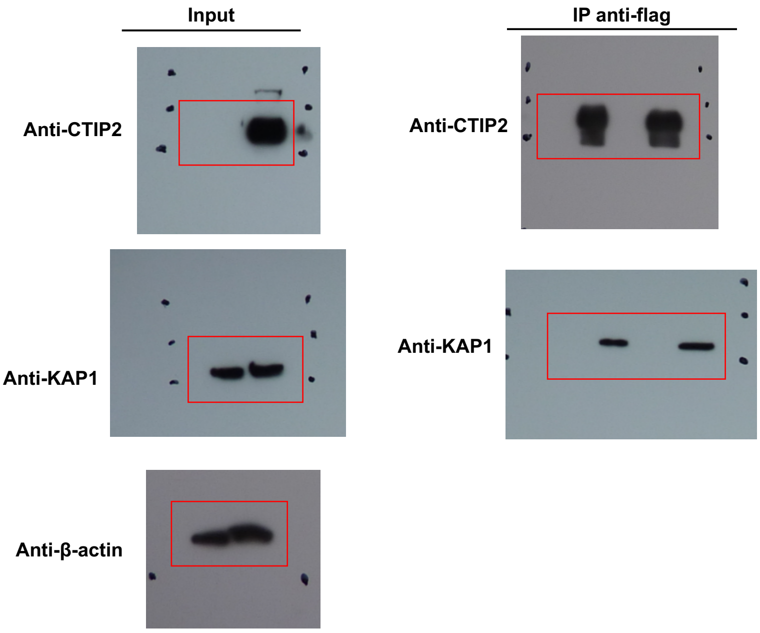

Figure 8B

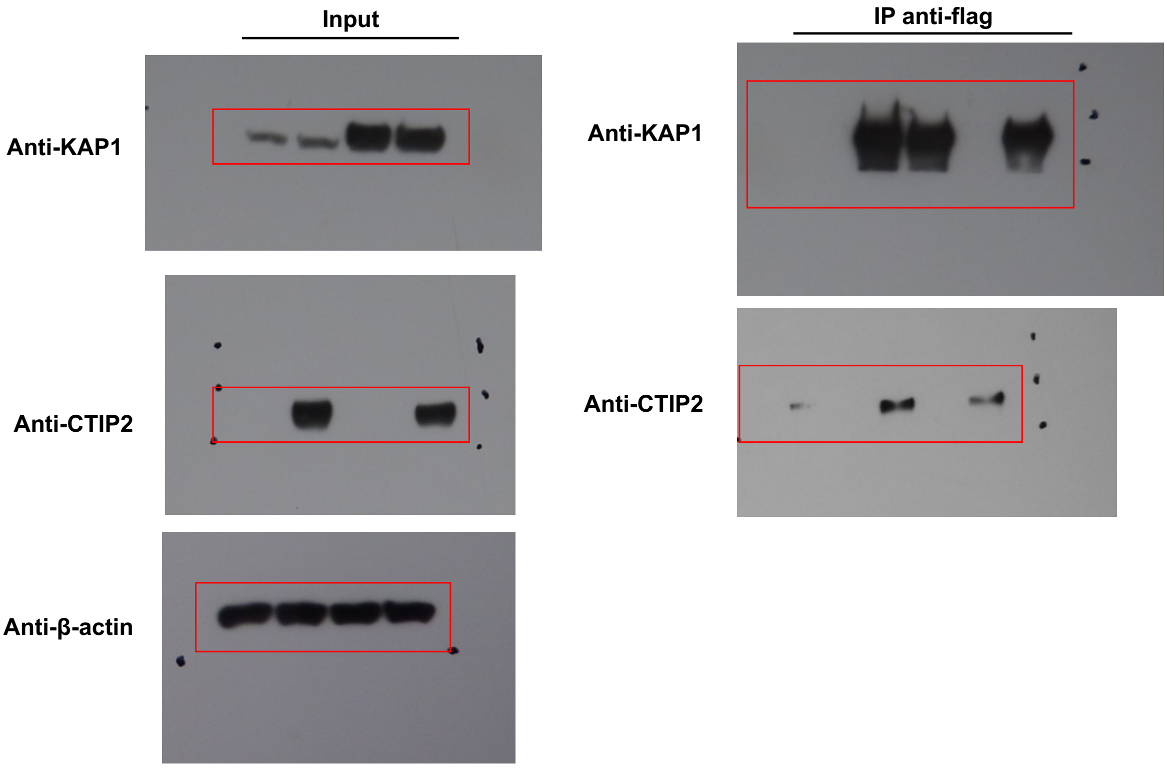

Figure 8C

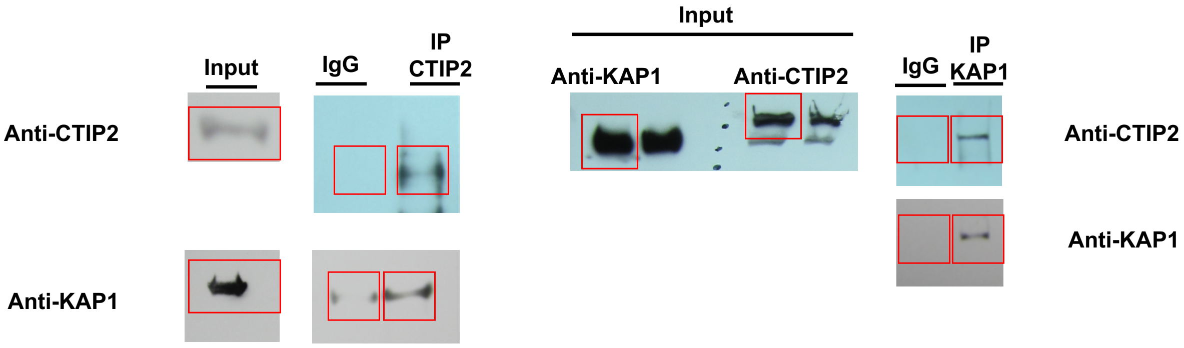

Figure 8D

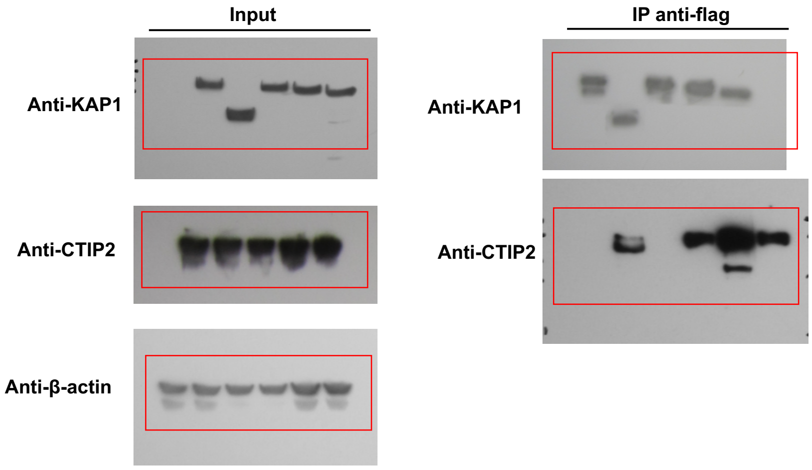

Figure 8E

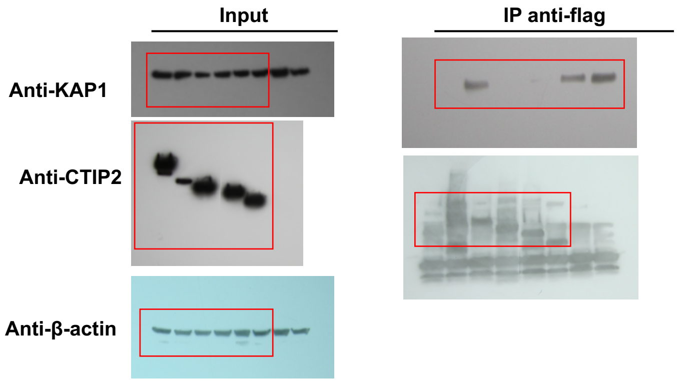

Figure 8G

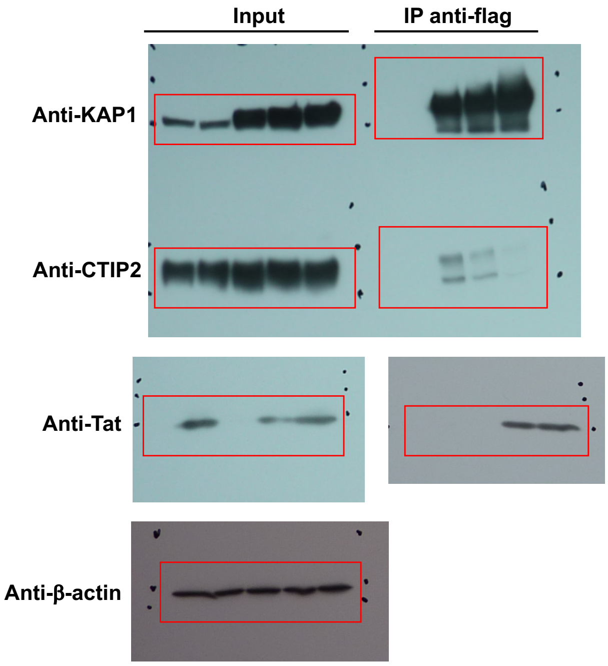

Supplement: Supplementary file 1 — Supplementary Information [file 41598_2021_82164_MOESM1_ESM.pdf]
